# Molecular Mechanism of the Piezoelectric Response in the β-Phase PVDF Crystals Interpreted by Periodic Boundary Conditions DFT Calculations

**DOI:** 10.3390/ma16176004

**Published:** 2023-08-31

**Authors:** Gianluca Serra, Alessia Arrigoni, Mirella Del Zoppo, Chiara Castiglioni, Matteo Tommasini

**Affiliations:** Dipartimento di Chimica, Materiali e Ingegneria Chimica Giulio Natta, Politecnico di Milano, Piazza Leonardo da Vinci 32, 20133 Milano, Italy; gianluca.serra@polimi.it (G.S.); alessia.arrigoni@polimi.it (A.A.); mirella.delzoppo@polimi.it (M.D.Z.)

**Keywords:** infrared, Raman, crystal polymorph, electrostatic interaction, Potential Energy Surface

## Abstract

A theoretical approach based on Periodic Boundary Conditions (PBC) and a Linear Combination of Atomic Orbitals (LCAO) in the framework of the density functional theory (DFT) is used to investigate the molecular mechanism that rules the piezoelectric behavior of poly(vinylidene fluoride) (PVDF) polymer in the crystalline β-phase. We present several computational tests highlighting the peculiar electrostatic potential energy landscape the polymer chains feel when they change their orientation by a rigid rotation in the lattice cell. We demonstrate that a rotation of the permanent dipole through chain rotation has a rather low energy cost and leads to a lattice relaxation. This justifies the macroscopic strain observed when the material is subjected to an electric field. Moreover, we investigate the effect on the molecular geometry of the expansion of the lattice parameters in the (**a**, **b**) plane, proving that the rotation of the dipole can take place spontaneously under mechanical deformation. By band deconvolution of the IR and Raman spectra of a PVDF film with a high content of β-phase, we provide the experimental phonon wavenumbers and relative band intensities, which we compare against the predictions from DFT calculations. This analysis shows the reliability of the LCAO approach, as implemented in the CRYSTAL software, for calculating the vibrational spectra. Finally, we investigate how the IR/Raman spectra evolve as a function of inter-chain distance, moving towards the isolated chain limit and to the limit of a single crystal slab. The results show the relevance of the inter-molecular interactions on the vibrational dynamics and on the electro-optical features ruling the intensity pattern of the vibrational spectra.

## 1. Introduction

Poly(vinylidene fluoride) (PVDF) is a widely used ferroelectric and piezoelectric polymer with well-assessed technological applications in several different fields [1,2,3,4,5,6,7,8]. The existence in PVDF of four different crystal polymorphs that exhibit different electrical activity makes its behavior complex. PVDF preferentially crystallizes in the nonpolar α phase (form II, TGTG′ conformation), which can be converted by stretching and poling to the polar β-phase (form I, all-trans conformation), where the piezoelectric response of the material is maximized. In addition to these two main phases, two other polar phases, γ (form III, T3GT3G′) and δ (form IV or polar α, TGTG′ conformation), can be generated, but they received less attention due to their much more elusive character and the lower piezoelectric response with respect to β-PVDF.

One of the more generally accepted hypotheses is that the piezoelectricity and pyroelectricity of the PVDF β-phase result somehow from the polar crystalline region of the polymer. Indeed, evidence that the orientation of dipoles in the crystalline phase occurs during the poling process has been published [9,10,11,12], but no conclusive evidence on the mechanisms responsible for polymer piezoelectricity at the microscopic level has been obtained [13]. Already at the beginning of the 1980s, Furukawa et al. [14] showed that the spontaneous polarization of this polymer was reversed by an external electric field of the order of 100 MV/m. Since then, many experimental studies supported this finding, and many theoretical models have been proposed; however, the exact mechanism at the basis of the piezoelectric behavior of PVDF has remained quite elusive since the discovery of this polymer [1]. In particular, little insight has been reached about the role of molecular interactions in stabilizing the piezoelectric state.

For all these reasons, in this work, we focus on the crystalline β-phase of PVDF, and we try to elucidate the role of intermolecular interactions in explaining the mechanisms that are responsible for the spontaneous reversal of the PVDF polarization under the action of an external electric field, keeping the level of our description as simple as possible. The real structure of PVDF is much more complex than the ideal β-phase crystal because different phases can coexist, and defects and amorphous regions are always present, thus complicating the interpretation of the experimental data.

To avoid these difficulties, we rely on high-level DFT calculations, which can provide reliable information on the energetics of the chain rotation, the role of the most important intermolecular interactions, and the modifications of the crystal structure induced by the chain rotation. Within the unavoidable approximations that we had to accept, we believe that this theoretical approach can grab the main features of the piezoelectric mechanism, leaving to subsequent works the analysis of further effects.

**Figure 1:**
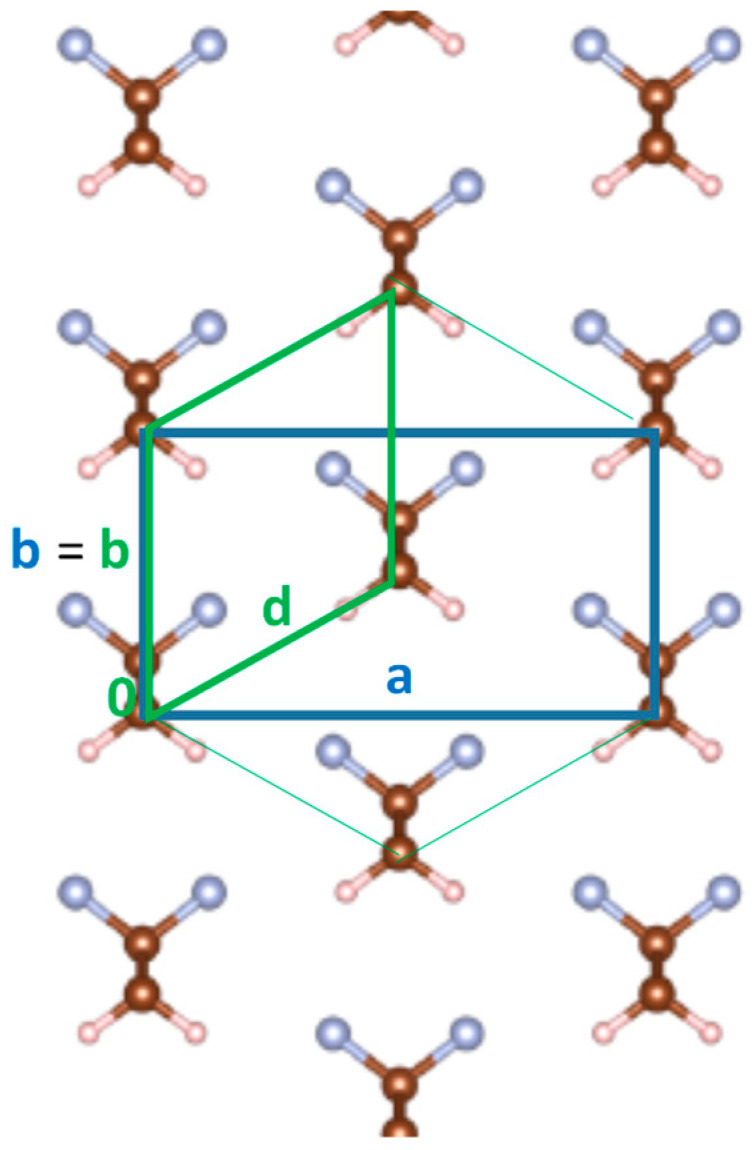
PVDF Crystal (β-phase): sketch of the face-centered orthorhombic unit cell (blue lines) in the (**a**, **b**) plane and of the primitive monoclinic unit cell (green lines) with the associated **b**, **d** axes. Color code for the atom’s description: gray = fluorine; brown = carbon; light brown = hydrogen. The thin green lines highlight the pseudo-hexagonal structure.

It must be noticed that in the β-phase crystal structure (i.e., orthorhombic with two trans-planar chains per cell), the dipole moments of the monomer units are aligned in the crystal so that a rigid rotation of the chain corresponds to the rotation of the dipole moment (see Section 3.1 and Figure 1). In this way, in the β-phase of PVDF, polarization reversal requires a 180° rotation of each chain. Kepler and Anderson [15] proposed a model where this reversal occurs by three rotation steps of 60°. The Kepler–Anderson model relies on the observation that the orthorhombic structure of the β-phase results from a small (1%) distortion of an underlying hexagonal primitive lattice, where the separation between molecules in the direction of the dipole moments is slightly smaller. Using the Kepler–Anderson model [15] as a starting point, we have investigated by Periodic Boundary Conditions (PBC) density functional theory (DFT) the rotational barriers of the chains in the crystal and the effect of the orientation of the chains on the deformation of the unit cell. By this theoretical approach, we can highlight the importance of electrostatic intermolecular interactions between hydrogen and fluorine atoms of nearby chains in determining the structural modifications under the action of external electric fields or applied deformations.

As a further probe for the study of intermolecular interactions, we will use theoretical vibrational spectra. The spectra evolution, as we progressively reduce the strength of the inter-chain interactions thanks to a controlled expansion of the cell parameters and the comparison with experimental data, will prove that intermolecular interactions are mandatory in determining the properties of the PVDF chains. In particular, isotropic expansions of the crystalline structure and expansions along preferential directions identified by the direction of the unit cell vectors will underline the most important intermolecular interactions determining the structural changes involved in the piezoelectric effect.

## 2. Materials and Methods

### 2.1. Materials

All the materials have been purchased from Sigma Aldrich (Schnelldorf, Germany) and used without further treatments. Poly(vinylidene fluoride) (PVDF) solutions, starting from PVDF in powder form with Mw = 534,000 g/mol, were dissolved in a (4:6) volume ratio of *N*,*N*-dimethylformamide/acetone (DMF, anhydrous (99.8%)/acetone (99.9%)) at a polymer/solvent concentration of 20% *w*/*v*. The solution was mixed using a magnetic stirrer at room temperature for about 1 h until a uniform and clear solution was obtained. Films have been prepared by drop-casting, homogeneously spreading the solution on the substrate, and completely evaporating the solvent by keeping the film 1 h under dynamic vacuum conditions.

### 2.2. Characterization

#### 2.2.1. FT-IR Spectroscopy

The IR absorption spectra were recorded using a Thermo Nicolet NEXUS FT-IR spectrometer (4 cm^−1^ resolution, 128 scans) equipped with a Thermo Electron Corporation Continuμm FTIR Microscope. Film spectra were recorded in transmission mode after depositing the solution on silicon (Si) substrates.

#### 2.2.2. Raman Spectroscopy

The Raman spectra were recorded with a Jobin Yvon Labram HR800 Raman spectrometer coupled to an Olympus BX41 microscope using a 50× objective. The excitation line of a diode-pumped solid-state laser (532 nm) was used with a power of 30 mW. Each spectrum was obtained as the average of 3 acquisitions of 40 s. Samples have been deposited on aluminum substrates for Raman inspection.

### 2.3. Computational Details

All DFT calculations were carried out within Periodic Boundary Conditions in the framework of the MO = LCAO approach by using the CRYSTAL17 code [16,17]. The choice of the functional and basis set, namely PBE0/pob-TZVP with no Grimme corrections for dispersion interactions, has been discussed in [18], where different methods have been compared, and the best computational methods proved to be PBE0/pob-TZVP, also in agreement with reference [19]. Notably, the PBE0 functional gives a good description of the crystal structure of all PVDF polymorphs, and the extended pob-TZVP basis set, explicitly optimized for CRYSTAL, is needed because an accurate description of intermolecular effects is required [20]. Indeed, the contribution of supramolecular electrostatic interactions is essential to obtain a good agreement between theoretical and experimental vibrational spectra [18]. Therefore, an accurate description of intermolecular interactions is also required for investigating the modulation of ferro- or piezoelectric properties of PVDF where non-covalent bonds play a central role.

## 3. Results and Discussion

The crystal structure of form I of poly(vinylidene fluoride) (PVDF) has been reported by several authors [21,22,23]. Lando et al. [23] determined the crystal structure of the β-phase by X-ray and wide-line NMR analyses. According to their result, two planar zigzag chains pass through the Cm2m orthorhombic unit cell. However, an “alternately-deflected” [24,25] molecular structure, or a statistically disordered packing of such deflected chains, was postulated to release the steric hindrance between the fluorine atoms along the chain. Because our work focuses on the role of intermolecular interactions and because the proposed chain deflections are very small, we will consider trans-planar chains as a first approach. This allows us to maintain the description of the chain rotation involved in the piezoelectric behavior as simply as possible. Successive investigations could address the study of the effects due to the slight chain deflections. However, a preliminary calculation on a PVDF crystal with alternately deflected chains does not show major changes, at least as far as the energetics is concerned.

In Figure 1, we report the conventional orthorhombic unit cell of the β-phase of PVDF with two chains per unit cell. **c** is the chain axis. Each monomer unit contributes to the total dipole with a dipole moment along the **b**-axis, mostly ascribed to the strong polar character of CF and CH bonds, which results in a negative fractional charge associated with fluorine and in electron-poor hydrogen atoms, carrying a fractional positive charge. For instance, according to [26], the electrostatic charges have been estimated as q_F_ = −0.22e and q_H_ = +0.18e.

It can be immediately seen that the orthorhombic cell results from a slight distortion of a hexagonal cell, where **b** and **d** (half the diagonal of the orthorhombic cell) are very similar in length: indeed, in the β-phase of PVDF they differ only by 0.6%. This slight deformation of the hexagonal cell decreases the distance between molecules in the direction of the dipole moment, as was already noticed by Kepler and Anderson [15].

Our calculations yield the following lattice constants of the orthorhombic cell (experimental data [22] in italics):

a = 8.478 (*8.58*) Å; b = 4.737 (*4.91*) Å; c = 2.561 (*2.56*) Å

According to the computed values, the length of half the cell diagonal is d = 4.856 Å, and the angle between **b** and **d** is 60.8°.

The underlying hexagonal symmetry suggests that a rigid rotation of the chains by 60° can be accommodated with only a slight deformation of the unit cell; we will show that the energy required for this rotation is quite small. In Figure 1, we also report the definition of the primitive cell of PVDF. We will refer to the primitive cell when considering the expansion of the crystalline structure along specific directions to study the relative importance of intermolecular interactions in different directions. Additionally, in this case, **c** is the chain axis, **b** is the polar axis, which coincides with the polar axis of the orthorhombic cell, whereas **d** is the oblique axis.

### 3.1. Energy Landscape for Chain Rotation

**Figure 2:**
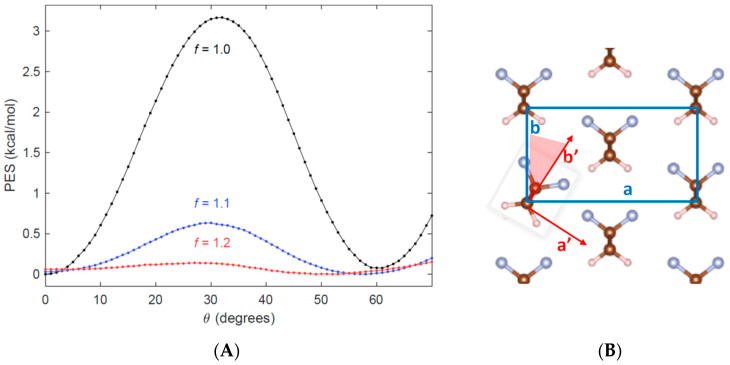
(**A**) Potential Energy Surface (PES) associated with the rigid rotation of the PVDF chain in the orthorhombic crystal unit cell. θ measures the dihedral angle between the chain plane and the crystallographic (**b**, **c**) plane of the orthorhombic cell (**c** is the vertical, chain axis). The PES is computed at different expansion factors f, starting from the optimized cell parameters f = 1.0 (black), f = 1.1 (blue), and f = 1.2 (red). The zero of the energy scale is set to the energy of the optimized crystal geometry, obtained for θ = 0°. Each point represents a value of the energy computed with a single point calculation; segments join neighboring points for better data visualization. (**B**) Sketch illustrating the rigid rotation of a PVDF chain and the definition of the θ angle (highlighted in light red). PBC guarantees that all the chains in the crystal have been rotated.

To show that the energy required for the rotation of the chains is quite small, we calculated the Potential Energy Surface (PES) along the θ coordinate, where θ is the angle defined by the plane containing the trans-planar CC chain skeleton and the direction of the polar axis **b**. The results are reported in Figure 2 and nicely meet the expectations of the Kepler–Anderson model [15]: the rotational barrier is only 3 kcal/mol, and a new minimum is found at about 60°.

**Figure 3:**
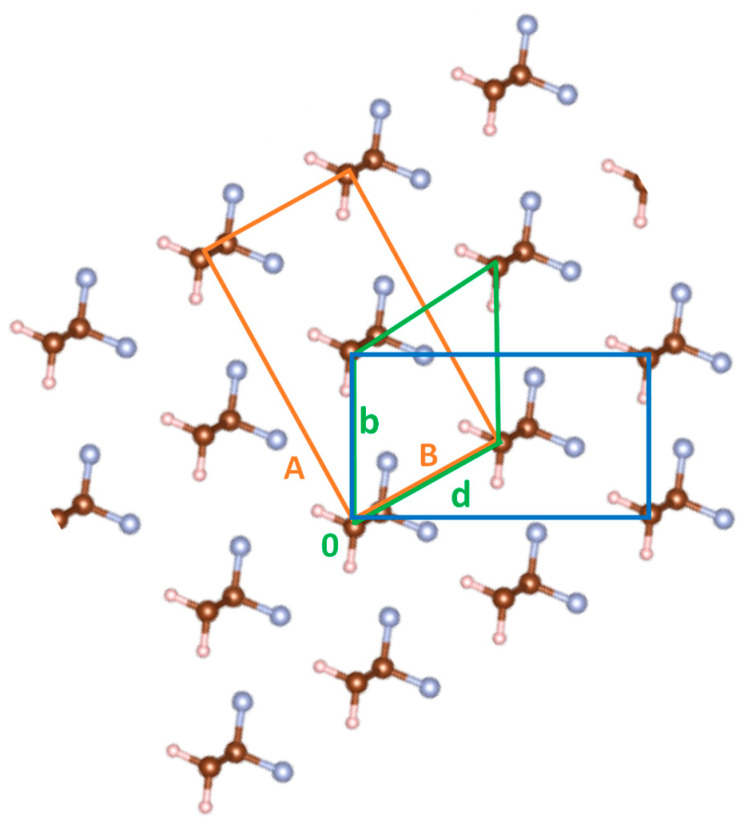
PVDF crystal structure (β-phase) as obtained by a rigid rotation of the chains with respect to the **b**-axis of the “original” orthorhombic chain (blue lines). After geometry optimization, the crystal structure relaxes according to the new orthorhombic cell (orange lines) with B = b and A = a. The new structure is attained starting from a structure obtained by a rigid chain rotation θ = 60°, but also starting from a structure with θ = 40° (in the last case, after optimization, we observe a further chain rotation of an additional 20° and the relaxation of the A, B cell parameters).

In Figure 2, we also show that the height of the barrier can be further reduced by increasing the intermolecular spacing by an isotropic expansion of the crystal in the (**a**, **b**) plane, obtained by multiplying the a, b cell parameters by the same factor f. Even if these expansions are sizeable (10% and 20% of the original cell dimensions), we obtain a clear indication that we can favor the chain rotation by increasing the intermolecular distances. Interestingly, it has been reported in the literature that increasing temperature enhances the piezoelectric response of PVDF and its copolymers [27], which is consistent with our observation on the PES. Our calculations have been done without re-optimization of the cell parameters and keeping the internal degrees of freedom of the chain fixed (rigid PES scan); this implies that a rigid rotation of the chains of 60° aligns the dipoles along the diagonal of the orthorhombic cell, namely the **d**-axis of the primitive cell (see Figure 3). The geometry obtained by means of this rigid rotation results in an intermolecular distance of the chains aligned along **d** that is slightly larger than the chain distance along the axis **b** (which coincides with the optimized b value of the electric axis of the “original” orthorhombic crystal). A relaxation of the cell parameters induced by the rigid rotation should recover the equilibrium relative positions, decreasing the distance between chains in the direction of the new polar axis and increasing the distance between chains along the other axis. Calculations prove indeed that this is what happens when, after the rigid rotation of the chain, we optimize the cell parameters. The mechanism is illustrated in Figure 3, showing the new orthorhombic cell (orange lines, **A**, **B** axes), which describes the new crystal structure obtained after the rotation of 60° of the PVDF chains.

The same mechanism of dipole rotation can be further applied to the new crystal geometry by subsequent rotation steps of 60°, thus obtaining a set of five new crystal structures (θ = 120°, 180°, 240°, 300°) corresponding to equivalent minima of the PES. Each step requires to overcome a PES barrier identical to that at about θ = 30°, occurring at θ = 90°, 150°, 210°, and 270°. As already pointed out in [15], three rotation steps of 60° determine the reversal of the dipole.

A further piece of evidence that chain rotations by 60° steps are plausible has been obtained as follows. By performing a full geometry optimization of a starting geometry where the chains in the orthorhombic cell are rotated by 40°, thus overcoming the potential barrier, a 3D lattice, rotated by 60°, is obtained, where the lattice constant in the direction of the new polar axis (**B**) equals in length that in the original cell (**b**), as described by Figure 3.

Such relaxation of the primitive cell after the rotation can explain the mechanism underlying the piezoelectric effect. Moreover, one might suppose that the rotation of one chain can drive the rotation of the neighboring chains to reduce the hindrance to the rotation, lowering the rotational barrier [28]. In this way, the whole crystalline domain is rotated.

If we now consider a crystalline domain with the polar axis **b** oriented in the z direction (in the lab reference system) and we apply an electric field that makes an angle larger than 30° with z and strong enough to produce the work on the dipole necessary to overcome the barrier, the chains can rotate and relax in the minimum at about 60°. As discussed above, the relaxation of the cell leads to shrinkage in the direction of the original oblique (**d**) axis (which is now the polar axis) and to the stretching along the z direction, which is needed to preserve the cell volume. In this way, a macroscopic deformation is obtained, i.e., the inverse piezoelectric effect. According to our model, the effect of the electric field is not spontaneously removed after the field is switched off because the new geometry attained corresponds to a new minimum of the PES.

When one crystalline domain is rotated, the surrounding domains can rotate in a coordinated way, but the details of the mechanism should be investigated in a dedicated work. For the time being, we suggest that the presence of defects, either conformational defects such as those present in the other PVDF phases (namely, α, γ, and δ) or line defects between different orientations of the domains, might act as the seeds of rotation. The role of amorphous regions should also be considered. Their presence might favor the rotation of small crystalline domains under the action of external electric fields.

**Figure 4:**
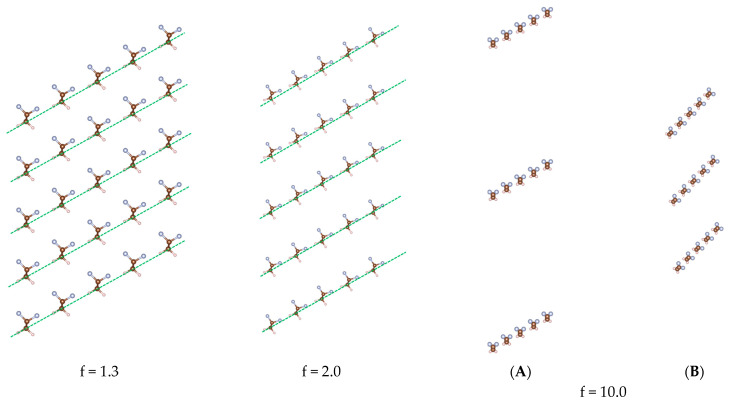
PVDF crystal structures obtained after cell expansion along the **b**-axis of the primitive cell of the β-phase. At the two expansion parameters f = 1.3, 2.0, the atoms’ coordinates have been optimized with frozen lattice parameters. Panels (**A**,**B**) describe the result of the complete optimization (cell parameters + atoms coordinates) starting from the expansion of the “original” crystal structure with f = 10 (**b′** = 10 × **b**) (**A**): after optimization, the chains are rotated of 60° and align along the **d** direction (**B**).

Finally, we suggest a possible mechanism for the direct piezoelectric phenomenon, that is, the induced polarization in response to an applied mechanical deformation. To do this it is useful to know the direction along which the most important electrostatic intermolecular interactions occur to do this. To this purpose, we separated the chains in the crystalline lattice by expanding the primitive cell along the polar axis **b** by applying an expansion factor f. In this way, we create progressively separated oblique slabs, namely separated (**d**, **c**) planes; in which the dipole moments make an angle of about 60° with the **d** direction. By expanding the cell along the **b**-axis, we weaken the H…F interactions between the chains aligned in the **b** direction. The optimization of the atomic coordinates, while keeping constant the increased distance between adjacent slabs, shows the tendency of the chains to rotate in such a way as to align their dipole moment towards the oblique direction of the **d**-axis—see Figure 4, cases f = 1.3, 2. In this way, the H…F interactions between nearby chains of the slab are at least partially recovered.

When the expansion is very large (f = 10), and the cell parameters are optimized together with the atomic coordinates, calculations show that a complete chain rotation (θ = 60°) is obtained (see Figure 4, f = 10.0). Not only do the chains orient themselves along the **d** direction, but adjacent chains, aligned along **d**, are also drawn closer. Indeed, after optimization, their distance decreases from 4.856 Å to 4.652 Å: this is even shorter than the b parameter of the optimized orthorhombic cell. Interestingly, after optimization, the distance between adjacent slabs decreases from the initial value (Figure 4B). This implies that long-range interactions, which the calculations might overestimate, are present. This overestimate of the intermolecular interactions might justify the exceedingly large expansions that are needed to rotate the chain by θ = 60°.

Even if the expansions to obtain the chain rotation by 60° are unphysical, the results fully support the basis of the Kepler–Anderson model [15] of chain rotation. A deformation along a preferential direction that separates the chains and weakens the intermolecular interactions, as in the case of expansions along the **b**-axis, may assist the reorientation of the dipoles and, hence, justifies the direct piezoelectric effect.

### 3.2. Vibrational Spectra

Vibrational spectroscopy played an important role in characterizing PVDF and PVDF-based systems [18,21,25,29,30,31,32,33,34,35,36,37,38,39]. Several authors provide experimental band assignment and characteristic marker bands of the different crystalline polymorphs by means of normal modes analysis based on empirical vibrational force fields [21,31,33] and, more recently, quantum mechanical predictions [18,38,39]. Vibrational frequencies have been obtained for isolated 1D chain models or 3D crystalline structures. We present here a discussion of the vibrational spectra of the β-phase of PVDF aimed at investigating the role of intermolecular interactions in modulating the vibrational dynamics and the associated dipole and polarizability derivatives that govern IR and Raman intensities.

The vibrational frequencies of the **q** = **0** optical phonons of the 3D crystal (15 modes) and of the 1D crystal (14 modes) at the Γ-point of the First Brillouin Zone have been calculated and classified according to the irreducible representations of the C_2v_ point group. The lowest frequency phonon of the 3D crystal, which describes the rotational mode of the individual chains around the **c**-axis, vanishes in the 1D crystal because it becomes free rotation.

**Figure 5:**
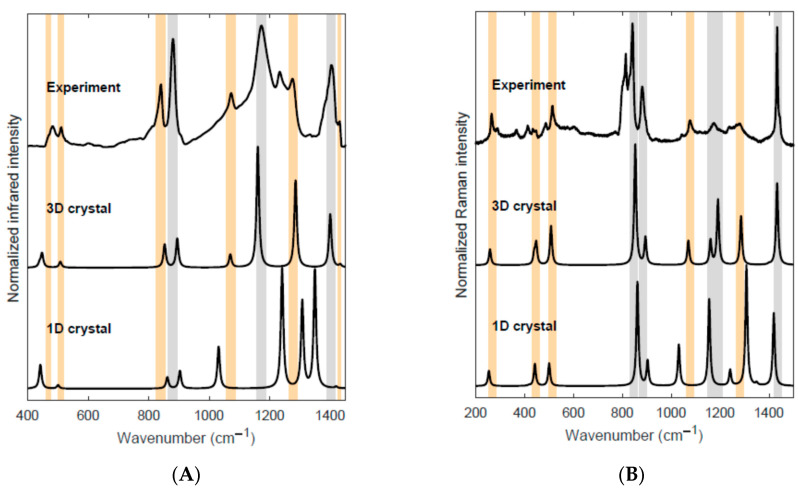
Panel (**A**). Experimental IR spectrum of a PVDF film with high β-phase content; DFT (PBE0/pob-TZVP) predicted IR spectrum of the 3D crystal and for an isolated all-trans PVDF chain (1D crystal). Panel (**B**). Experimental Raman spectrum (λ_exc_ = 532 nm) of a PVDF film with high β-phase content; DFT (PBE0/pob-TZVP) predicted Raman spectrum of the 3D crystal and for an isolated all-trans PVDF chain (1D crystal). Theoretical Raman band intensities are obtained as $I_{k}^{Raman\;}=A_{k}^{Raman}/\nu_{k}$, where $A_{k}^{Raman}$ is the computed Raman activity and $\nu_{k}$ is the vibrational wavenumber of the k-th normal mode. The computed peak wavenumbers have been scaled by empirical factors (see text) of 0.9892 (3D crystal) and 0.9742 (1D crystal). The orange stripes highlight the experimental bands, which can be considered spectroscopic markers of the PVDF β-phase. The bands highlighted by grey stripes correspond to vibrational transitions of the PVDF β-phase, showing a frequency close to peaks of the α-phase and/or of the amorphous phase: these bands possibly hide contributions due to phases different from the β one.

Figure 5 and Table 1 allow the comparison between theoretical predictions and the experimental IR and Raman spectra, which have been recorded on a PVDF film characterized by a dominant content of the piezoelectric β-phase. The presence of the crystalline β-phase is proven by the observation of its marker bands highlighted in orange in Figure 5 and identified by a (*) in Table 1. Some other bands, assigned to the β-phase but with peak wavenumbers close to characteristic peaks of a different crystalline phase and/or the amorphous phase, are highlighted in grey in Figure 5. Figure S1 of Supplementary Materials reports a comparison between the experimental spectra of a PVDF film (same spectra reported in Figure 5) and the spectra of a fibrous PVDF sample showing a large amount of α crystals. In the spectra of PVDF fibers, we clearly observe several marker bands of the α-phase, which instead are barely detected in the spectra of the PVDF film, thus proving that the contribution of the β-phase is dominant in this sample. However, because of the presence of some crystal domains of the more stable α-phase and possibly because of the presence of other crystal polymorphs, the experimental spectra are much more structured than those obtained by the theory, which describes a defect-free β-crystal in the β-phase. In addition, the unavoidable presence of polymer chains belonging to the amorphous phase contributes to the vibrational spectra with broad features. Figure S1 of Supplementary Materials further illustrates how the presence and the different amount of several crystal polymorphs in PVDF samples affects their spectral pattern. These complex experimental features make the comparison of the experimental band intensity pattern with the computed IR and Raman intensities complicated.

To overcome this difficulty, we adopted a band deconvolution procedure to obtain an estimate of the relative IR and Raman intensities of the band components associated with the β-phase. The details about the results of the curve fitting, carried out with the Fityk software, are illustrated in the Supplementary Materials, Figures S2 and S3, while the experimental relative intensities of the individual bands obtained from this analysis are reported in Table 2, together with the IR and Raman intensity data obtained from DFT calculations (3D and 1D model).

A scaling procedure of the predicted wavenumbers reported in Table 1 has been carried out by adopting empirical scaling factors obtained from the linear correlation coefficient between experimental and theoretical frequency data. For the normal modes observed both in the Raman and IR spectra, the average wavenumber is used. Table 1 reports the scaled frequencies of both the 3D crystal and the isolated (all-trans) chain (1D crystal): the scaling factors have been determined independently for the two models, and they are respectively 0.989 and 0.974 for the 3D and the 1D crystal. In the scaling procedure, we did not include the CH stretching modes because of their well-known remarkable anharmonicity, which would result in lower scaling factors and too large a downshift of the other frequencies.

The use of an independent scaling factor, which guarantees the best fit of the experimental frequencies both with the 1D and with the 3D models, allows us to highlight the remarkable role of the intermolecular interactions in the vibrational dynamics of PVDF. Indeed, the prediction in the spectral region between 1000 and 1400 cm^−1^ shows several shortcomings of the 1D model, with differences of 40–50 cm^−1^ between predictions and experiments. Instead, the quality of the frequencies predicted by the 3D model is always excellent, with relative errors which, in most cases, do not exceed 1%.

According to the 3D model, the highest frequency A_2_ band is predicted at 1190 cm^−1^, showing a rather large Raman activity—about two times that of the B_2_ transition at 1060 cm^−1^. Following this indication, the early empirical assignment [21] of the A_2_ transition to a very weak Raman feature observed at 980 cm^−1^ should be revised. We suggest that the broad band observed at about 1170 cm^−1^ is due to the convolution of the A_2_ band and of a B_2_ transition, as indicated in Table 1 and Table 2.

The IR intensities predicted for the single chain are remarkably different from those of the 3D model. In particular, considering total IR intensity and Raman activity, obtained as the sum over all vibrational transitions, there is an increase by a factor of about 2 (IR) and 3.5 (Raman) going from the 1D to the 3D model (see Table 2, last row). The enhancement of vibrational intensities due to the dielectric environment in the crystalline phase has already been discussed in the literature [40]; however, changes in the intensity pattern with important variations of internal intensity ratios are to be mainly ascribed to specific intermolecular interactions which affect in different ways each normal mode. Phenomena of this kind are well-known and very important in the presence of strong intermolecular interactions, such as hydrogen bonds [41,42,43].

It is evident that the strong electrostatic interactions between the electron-poor hydrogen atoms and the negatively charged fluorine atoms of nearby PVDF chains well-packed in the β-crystal play a major role in determining the intensity pattern in the region 1000–1500 cm^−1^. This feature parallels the effect of the intermolecular interactions on the vibrational dynamics, as proven by the remarkable differences in the vibrational frequencies of 3D and 1D models in the same region.

Even if the normalized intensities obtained by the experiments suffer limitations due to some arbitrariness in the curve fitting procedure applied to broad and structured bands, the comparison between experiments and theory (Table 2—last six columns) further proves that the 1D model does not capture the main experimental features. The 1D model predicts two dominant IR transitions (at 1241 and 1349 cm^−1^) about 10 times stronger than the reference band, namely the internal intensity standard at 861 cm^−1^; instead, the 3D model predicts that the strongest IR bands occur at 1160, 1285, and 1399 cm^−1^. The most intense band (1160 cm^−1^) is about five times stronger than the reference band, in good agreement with the experiment. Even if the band calculated at 852 cm^−1^ and the band at 1399 cm^−1^ are underestimated and overestimated, respectively, by the calculation for the 3D crystal and considering the difficulties related to the band deconvolution, which hinder accurate quantitative evaluations, it is clear that the 3D model better predicts the IR intensity pattern, while the 1D model dramatically fails. Boldface characters in Table 2 highlight normalized intensity values, which are from 5 to 10 times larger than experimental observation.

Moreover, the predicted Raman intensity pattern of the 3D model fits the experimental observation better than the intensity pattern of the 1D model. For instance, notice modes 8 and 11, whose relative intensities in the 1D model are remarkably larger than those of the experiment and the 3D model.

In conclusion, the careful comparison with the experimental spectra clearly shows that the isolated chain model cannot be adopted for a reliable prediction of both the vibrational dynamics of PVDF and the associated IR and Raman intensities. This point had already been qualitatively observed by comparing experimental and theoretical spectra [18], and the quantitative comparison presented here consolidates this finding. This observation is particularly relevant in the framework of the theoretical modeling of polymeric materials, which often makes use of single chains “in vacuo” as models for the study of their structure and vibrational frequencies, even when strong and specific interchain interactions take place.

**Figure 6:**
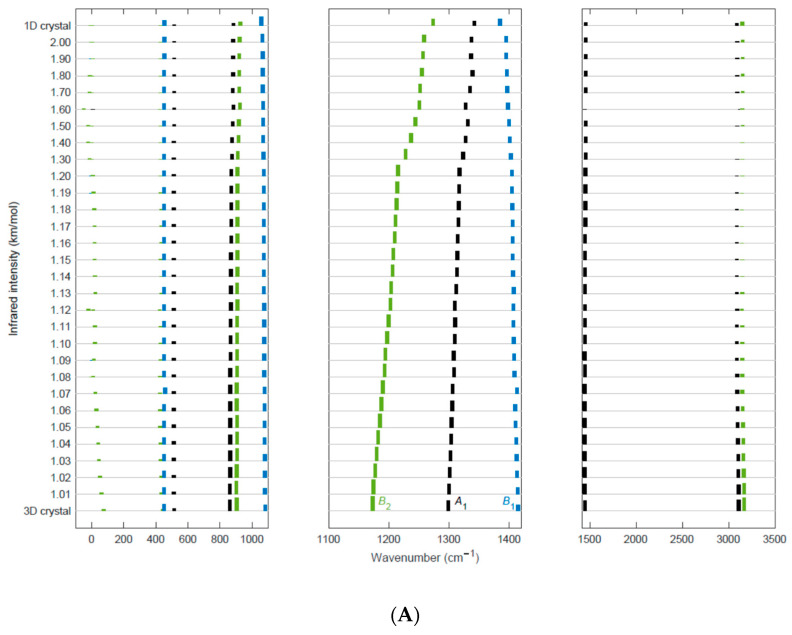

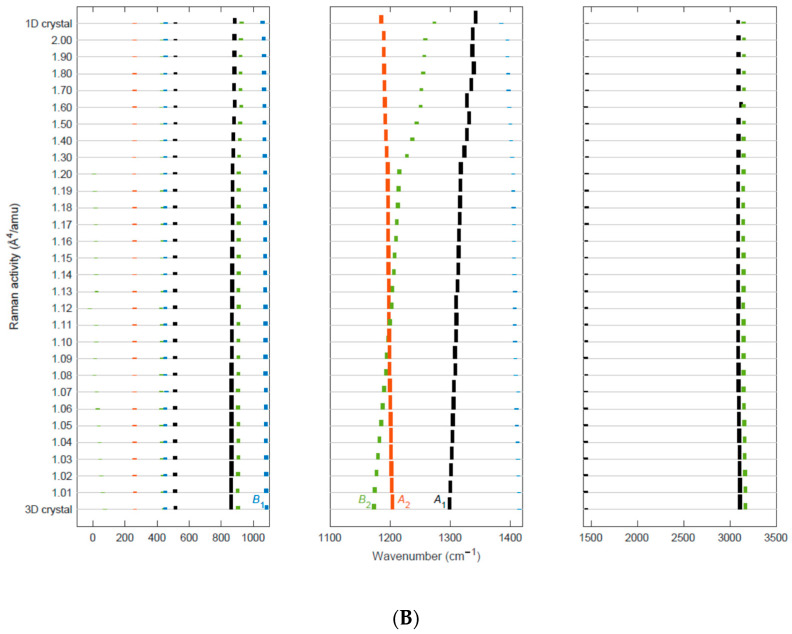
Predicted IR (**A**) and Raman (**B**) spectra of the β-crystal of PVDF for different values of the f parameter, describing the isotropic lattice cell expansion in the crystallographic (**a**, **b**) plane (see Figure 1 for the definition). Each spectrum is represented by a histogram with bar heights proportional to the computed IR intensities (or Raman activities). The spectra are stacked and vertically shifted according to the f parameter (ranging from f = 1, corresponding to the optimized 3D crystal geometry to f = 2). On top of each panel, the spectrum of the isolated chain (1D crystal, trans-planar chain conformation) is reported. A color code classifies the peaks according to the suitable irreducible representation of the C_2v_ point group (A_1_: black, A_2_: red; B_1_: blue; B_2_: green). Negative wavenumber values in the plot correspond to imaginary frequencies. Both IR and Raman spectra have been split into three different panels, which illustrate different spectral regions: for each region, a different normalization factor has been applied to the intensity values, while in a given panel the same factor has been applied to all the bands and all the spectra at different f values. In this way, it is possible to appreciate the intensity evolution of all the bands, including those very weak.

Figure 6 presents a computational test, which shows that the intermolecular forces in the PVDF β-crystal have long-range effects on the spectroscopic response of PVDF. The figure describes the evolution of the computed IR (Figure 6A) and Raman (Figure 6B) spectra with the isotropic expansion of the cell parameters a and b.

The vibrational spectra have been computed in the following way: by starting from the fully-optimized geometry of the orthorhombic PVDF crystal (β-phase), we obtain new lattice geometries by applying an expansion factor f to the cell parameters a and b, while the c parameter is kept fixed (hereafter we will label each new lattice geometry by its f parameter). f ranges from 1.01 to 2; finer steps of 0.01 have been used from f = 1.01 to f = 1.2, then steps of 0.1 have been adopted. For each expansion, new fractional atomic coordinates are obtained by applying the factor 1/f to those of the optimized crystal geometry, which corresponds to f = 1. This procedure allows to obtain expanded lattices, with intramolecular geometry identical to that of the f = 1 case. Moreover, the procedure guarantees that the chain orientation in the cell is maintained, with the plane of the trans-planar polymer backbone lying in the (**c**, **b**) plane. Each f-structure is used as initial guess geometry for performing optimization of the atomic positions with fixed cell parameters: after the optimization, the intramolecular geometry relaxes, while the lattice parameters remain frozen. The optimized chain geometries are used to calculate the IR and Raman spectra, shown in Figure 6. Values of the calculated frequencies and intensities for selected f values are reported in Supplementary Materials (Table S1).

Each spectrum is shown in Figure 6 as a histogram with bar heights proportional to the computed IR intensities (or Raman activities). A color code classifies the peaks according to their irreducible representation in the C_2v_ point group. Negative wavenumber values in the plot correspond to imaginary frequencies. They are included in the plot because they give interesting information concerning the evolution of the lowest frequency lattice mode of the 3D crystal, which describes the rotation of the individual chains around their axis (that corresponds to the **c**-axis of the crystal). The predicted wavenumber for this mode is very close to the experimental determination of 70 cm^−1^ [21]. Due to the nature of the nuclear displacements associated with this phonon (chain rotation), its wavenumber is a proxy of the curvature of the PES illustrated in Section 3.1, and its low wavenumber clearly indicates that this rotation mode is rather soft. Remarkably, the frequency of the rotation mode quickly decreases as the lattice expands; this behavior is expected because the intermolecular interactions are less effective as the chain distance increases. Furthermore, the frequency softening can be correlated to the decrease of the PES barrier for increasing cell volume (Figure 2). At f = 1.3, the rotational mode has a negative wavenumber (corresponding to the feature at −13 cm^−1^ in the plot), which reaches a large negative value (−49 cm^−1^) at f = 1.6, while between f = 1.8 and f = 2.0, it approaches zero, which corresponds to the free rotation of an isolated chain. For some values of f, two IR active modes with wavenumber close to zero can be spotted in Figure 6: this is an artifact resulting from the mixing of the rotational mode with the pure translation of B_2_ symmetry species.

The negative wavenumbers computed in the range 1.3 < f < 1.8 indicate that the crystal geometry with PVDF chains lying in the (**a**, **b**) plane (i.e., θ = 0) does not correspond to a well-defined minimum of the PES. Probably, this feature corresponds to a flat potential energy landscape as a consequence of the negligible energy barrier of V(θ) we computed already for f = 1.2 (see Section 3.1).

The largest frequency dispersion with f is observed for the modes in the 1100–1400 cm^−1^ region: in particular, the B_2_ mode at 1173 cm^−1^ (3D crystal, unscaled wavenumber) shifts by about 100 cm^−1^ at f = 2 and shows a continuous trend towards the frequency of the isolated chain. However, even at f = 2, this mode does not reach the limiting value of the 1D model.

Table 3 shows the total IR intensity and Raman activity given by the sum of band intensities over all normal modes. These values give a feeling of the remarkable intensity changes in the IR or Raman spectra with increasing f. Moreover, Table 3 reports the partial contributions to the total intensity from the modes of each symmetry species. The major changes in frequencies and intensities concern modes belonging to the B_2_ and A_1_ irreducible representation of the C_2v_ symmetry group. These modes are characterized by atomic displacements orthogonal to the chain axis, whereas B_1_ modes involve displacements along the **c** axis, which are less affected by the cell expansion in the plane orthogonal to **c**. This is another evidence of the inter-chain dynamical and electro-optical coupling, which depend on the distance between nearby chains and mainly affect the modes for which the atoms explore the inter-chain space, as in the case of A_1_ and B_2_ vibrations. For instance, the B_2_ band at 1173 cm^−1^ can be assigned to a normal mode, which is a combination of CF_2_ antisymmetric stretching and CH_2_ rocking [18,21].

An odd intensity behavior can be observed for the weak symmetric and antisymmetric CH_2_ stretching transitions (Figure 6). Both IR bands show a non-monotonic intensity trend with increasing f. At first, the intensities decrease and reach a vanishing value for f = 1.3–1.4. By further increasing f from 1.5 to 2, the intensities increase again and reach the value of the isolated chain.

**Figure 7:**
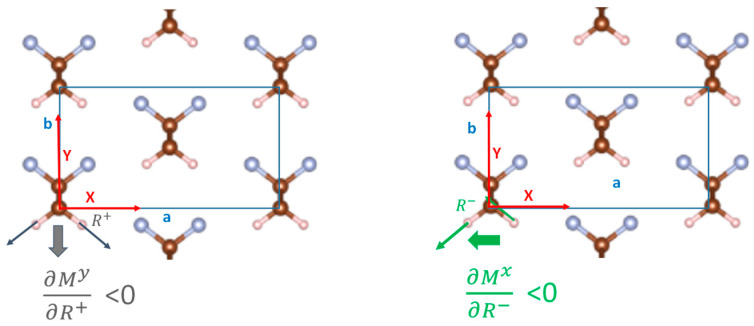
Sketch of the Q_14_ (symmetric stretching—left) and Q_15_ normal modes (antisymmetric stretching—right) of the PVDF β-crystal (the thin arrows describe the displacements of the H atoms, while the thick arrows the associated dipole moment variation, in the Y and X directions, respectively. For the sake of simplicity, the nuclear displacements are illustrated for just one asymmetric unit.

This behavior can be rationalized by considering the dipole derivatives associated with the symmetric CH_2_ stretching mode (Q_14_ = R+; A_1_ symmetry, dipole derivative along the **b**-axis) and with the antisymmetric CH_2_ stretching mode (Q_15_ = R−; B_2_ symmetry, dipole derivative along the **a**-axis) (Table 4). The reference axes and nuclear displacements are shown in Figure 7, which schematically illustrates, for the 3D model with f = 1, the negative dipole changes associated with positive displacements along the two stretching modes (A_1_, B_2_). In a simplified picture based on the theory of electro-optical infrared intensity parameters [44,45,46,47], describing the CH stretching dipole derivatives as due to local dipole moments associated with each CH bond, we can conclude that the local bond-dipole derivative $\left(\;\frac{\partial \mu_{CH}}{\partial r_{CH}}\;\right)$ is positive, while usually, sp^3^ CH bonds show $\frac{\partial \mu_{CH}}{\partial r_{CH}}<0$ [46,47,48]. Interestingly, as the cell expands, $\frac{\partial M^{y}}{\partial Q_{14}}$ and $\frac{\partial M^{x}}{\partial Q_{15}}$ decrease, and then they change their sign. This happens when going from f = 1.3 to 1.4 for Q_14_ (A_1_) and from 1.2 to 1.3 for Q_15_ (B_2_). Thus, approaching the case of the isolated chain, the sign of the dipole derivatives is reversed and can be rationalized by local parameters with the usual sign, i.e., $\frac{\partial \mu_{CH}}{\partial r_{CH}}>0$. A reasonable way to explain the anomalous behavior in the 3D crystal is to admit that, in the crystal, the sign of the dipole derivatives $\frac{\partial M^{y}}{\partial Q_{14}}$ and $\frac{\partial M^{x}}{\partial Q_{15}}$ cannot be determined just considering the local CH dipole derivatives $\left(\;\frac{\partial \mu_{CH}}{\partial r_{CH}}\;\right)$, but it is necessary to also consider the contribution of charge fluxes arising from the nearby chains [43]. Considering, for instance, the symmetric CH_2_ stretching, the hydrogen atoms that move closer to the F atoms of the adjacent chains could experience a negative charge flux from the F atoms, which could justify the negative sign of $\frac{\partial M^{y}}{\partial Q_{14}}$. A similar mechanism has been illustrated in the past in the case of hydrogen-bonded dimers, showing a partial charge transfer between the electron acceptor and the electron donor molecules, which is modulated by the stretching of the XH bond involved in the XH…Y hydrogen bond.

Following the same method adopted for the isotropic expansion of the cell in the (**a**, **b**) plane, we have analyzed the effects on the spectrum of an anisotropic cell expansion, obtained by increasing only the cell parameter a, by a factor f, thus going towards vertical slabs with chains in the (**c**, **b**) plane.

**Figure 8:**
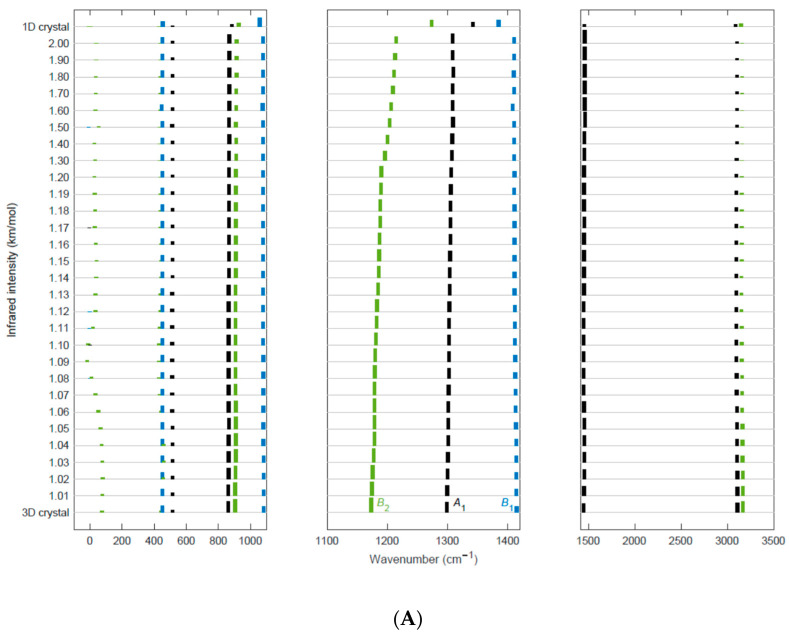

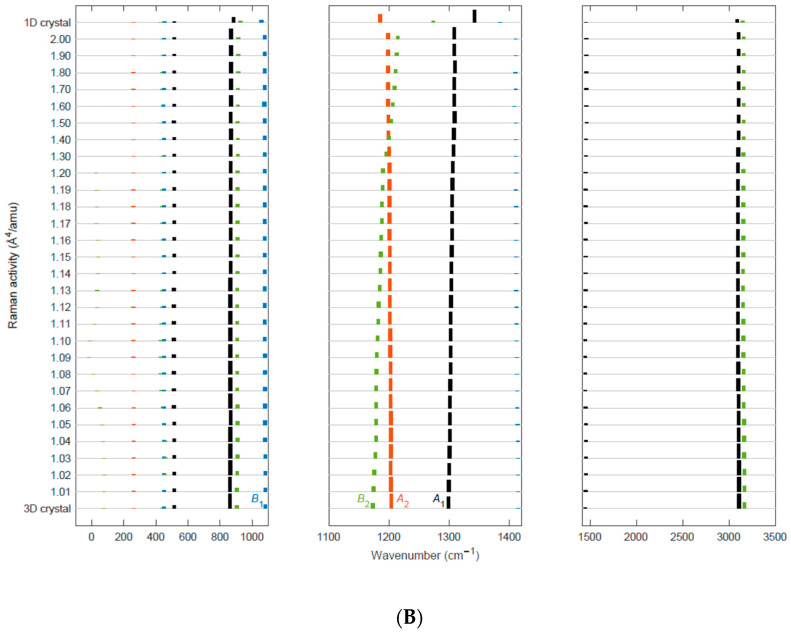
Predicted IR (**A**) and Raman (**B**) spectra of the β-crystal of PVDF for different values of the f parameter, describing isotropic lattice cell expansions along the **a** direction. Each spectrum is represented by a histogram with bar heights proportional to the computed IR intensities (or Raman activities). The spectra are stacked and vertically shifted according to the f parameter (ranging from f = 1, corresponding to the optimized 3D crystal geometry to f = 2). On the top of each panel, the spectrum of the isolated chain (1D crystal, trans-planar conformation of the chain) is reported. A color code classifies the peaks according to the suitable irreducible representation of the C_2v_ point group (A_1_: black, A_2_: red; B_1_: blue; B_2_: green). Negative wavenumber values in the plot correspond to imaginary frequencies. Both IR and Raman spectra have been split into three different panels, which illustrate different spectral regions: for each region, a different normalizing factor has been applied to the intensity values, while in a given panel the same factor has been adopted for all the bands and all the spectra at different f value. In this way, it is possible to appreciate the intensity evolution of all the bands, including the very weak ones.

Figure 8 describes the evolution with f of the computed IR (Figure 8A) and Raman (Figure 8B) spectra. Additionally, at larger f values, the spectra are far from the typical pattern of an isolated chain; this result is expected since strong inter-chain interactions are still present in the vertical slab at any f value. Moreover, the frequencies and intensities of all the modes except for some B_2_ modes are only slightly perturbed by the expansion of the cell in the **a** direction. Interestingly, at f = 2, the contribution to the total IR intensity of the A_1_ and B_1_ modes is about the same as that obtained for the 3D crystal, while the contribution of the B_2_ modes is less than half (see Table 3). A similar situation occurs with Raman spectra, which, however, show a non-negligible effect of the slab separation also on A_1_ transitions.

The CH_2_ stretching IR intensities do not show the non-monotonic behavior observed for the isotropic expansions, and this is further evidence that interchain electro-optical interactions play a role. However, the B_2_ band is much more sensitive to the expansion along **a**, a phenomenon which can be related to the fact that during the antisymmetric stretching, the hydrogen atoms are displaced in the direction of the **a**-axis, and the mode has a dipole derivative along **a** (x component, see Figure 7).

In the case of the isotropic cell expansion, the increasing separation of vertical slabs with increasing f affects the frequency of the rotational mode. At first, we observe a decreasing trend, reaching a small negative value of the frequency parameter at f = 1.1; then, the frequency value rises to 39 cm^−1^ for f = 2. It is clear that the softening of the rotational mode is associated with the decrease of the inter-chain interactions between chains belonging to different vertical slabs, showing once again that the energetics of the chain rotation are very sensitive to crystal strains.

In conclusion, the analysis of the computed vibrational spectra for the 3D and 1D models reveals strong inter-chain interactions between H and F atoms belonging to nearby chains of the same vertical slab and adjacent vertical slabs. Each F atom benefits from interactions with four H atoms belonging to two nearby chains. These interactions can be classified as due to electrostatic forces because H atoms carry positive partial charges and F atoms are negatively charged but reveal some charge flux features that are found in hydrogen-bonded systems. Such features are highlighted by the non-monotonic evolution of the CH_2_ stretching intensities with the crystal expansion.

## 4. Conclusions

In this paper, we have shown that density functional theory calculations within Periodic Boundary Conditions and the LCAO representation of the crystal orbitals (as implemented in CRYSTAL17) support the understanding of the molecular mechanisms that underlie the piezoelectric response of PVDF. Many factors contribute to the behavior of PVDF, and a detailed analysis is not straightforward. However, carefully designed theoretical “experiments” enable disentangling the different contributions to complex phenomena such as this one, primarily when experimental approaches cannot provide direct access to a single effect. Based on the well-documented assumption that the most significant contribution to the piezoelectric behavior of PVDF comes from the crystalline β-phase, we modeled the crystal structure of PVDF with an orthorhombic lattice with two trans-planar chains. The quasi-hexagonal structure of the crystalline lattice and the features of the PES obtained from DFT calculations suggest that it is quite easy to rotate the chains in the crystal by 60°, notably under the influence of an external electric field. Such a field-induced chain rotation causes a corresponding rotation of the dipole moments. This leads to a specific change in the dimension of the unit cell that relaxes and shrinks along the direction of the applied field. In this way, an electric field applied in the appropriate direction produces the deformation at the basis of the piezoelectric effect. Furthermore, DFT calculations also show that a mechanical deformation applied in the right direction may induce the rotation of the dipoles. Here, the driving force of the piezoelectric behavior is the weakening of intermolecular interactions caused by the applied deformation and the tendency to recover these stabilizing interactions along the new polar axis.

The central role of intermolecular interactions in allowing chain rotation has also been analyzed based on the simulation of the vibrational spectra of selected models of PVDF. By expanding the crystalline unit cell and monitoring the change in frequency and intensity in the infrared and Raman spectra, we could highlight the peculiar behavior of the lowest frequency lattice mode of the 3D crystal, which is assigned to the rotation of individual chains around their axis. The frequency of this phonon decreases as the lattice expands, and it becomes negative for an expansion factor f = 1.3 and finally approaches zero when it becomes the free rotation of an isolated chain. This points out the relevance of intermolecular interactions in modulating chain rotation in PVDF, which is at the basis of the Kepler–Anderson model of PVDF piezoelectricity [15]. As expected in systems with non-negligible intermolecular interactions, the experimental IR and Raman spectra can be reasonably reproduced by the DFT calculations only when a 3D model is used, while the single chain 1D model performs poorly.

The results presented here are a first-order approach to the description of the Kepler–Anderson mechanism of PVDF piezoelectricity. In spite of its simplicity, this approach can account for the essence of the phenomenon. Future investigations should consider the possible role played by intramolecular defects, line defects, domain boundaries, or the role of the amorphous regions. In this framework, additional insight into the molecular mechanisms leading to piezoelectricity in PVDF will require considering the characteristic times necessary for the material structure relaxation. These features are relevant in the perspective of applications that require fast responses to field oscillating at high frequencies.

Further DFT calculations, simulating the crystal structure relaxation in the presence of an external electric field, could give a quantitative indication of the contribution of the chain rotation mechanism to the individual piezoelectric constants of PVDF, hopefully supporting our conclusions about the relevant molecular mechanisms.

## Figures and Tables

**Table 1:** Wavenumbers of the fifteen **q** = **0** phonons of PVDF: DFT-PBE0/pob-TZVP calculated wavenumbers for the 3D crystal (Cm2m space group, point symmetry at Γ: C_2v_) and for the 1D crystal (single chain, point symmetry at Γ: C_2v_). DFT wavenumbers after scaling (see text) and experimental wavenumbers from the spectra of Figure 5. The symbol (?) indicates a tentative new assignment for the A_2_ transition, as suggested by DFT results. The symbol (*) highlights marker peaks of the β-phase.

Mode #	Wavenumbers (cm^−1^) DFT		Wavenumbers (cm^−1^) DFT, after Scaling		Wavenumbers (cm^−1^) Experimental	
	1-D	3-D	1-D	3-D	IR	Raman
1 (B_2_)		75		74	70 from Ref. [21]	-
2 (A_2_)	260	261	254	258	-	262 (*)
3 (A_1_)	429	444	418	439	-	445 (*)
4 (B_1_)	453	452	442	447	474 (*)	-
5 (A_1_)	514	513	500	508	510 (*)	513 (*)
6 (A_1_)	884	862	861	852	840 (*)	841
7 (B_2_)	927	904	903	894	880	880
8 (B_1_)	1058	1081	1031	1069	1072 (*)	1076 (*)
9 (B_2_)	1273	1173	1241	1160	1172	1172
10 (A_2_)	1185	1204	1155	1190	-	1172 (?)
11 (A_1_)	1342	1299	1307	1285	1275 (*)	1275 (*)
12 (B_1_)	1385	1415	1349	1399	1402	-
13 (A_1_)	1457	1448	1419	1432	1429 (*)	1432
14 (A_1_)	3091	3108	3011	3074	2976	2979
15 (B_2_)	3149	3167	3067	3132	3015	3018

**Table 2:** DFT-PBE0/pob-TZVP predicted IR intensity (km mol^−1^) and Raman Activity (Å^4^ amu^−1^) of the fifteen **q** = **0** phonons of PVDF 3D crystal and 1D crystal (single chain); intensity values normalized to the band intensity of the A_1_ transition at 840 cm^−1^ are reported and compared with intensity ratios from experiments. Boldface numbers highlight normalized intensity values from 5 to 10 times larger than the observed ones.

Mode #	IR Intensity (km mol^−1^)	Raman Activity (Å^4^ amu^−1^)	IR Intensity (km mol^−1^)	Raman Activity (Å^4^ amu^−1^)	Normalized IR Intensity			Normalized Raman Intensity		
	1D	1D	3D	3D	1D	3D	exp	1D	3D	exp
1 (B_2_)			9.79	0.01		0.17	-		0.01	-
2 (A_2_)	-	0.28	-	0.70	-	-	-	0.14	0.13	0.13
3 (A_1_)	0.29	0.02	9.73	0.80	0.02	0.17	-	0.01	0.09	0.03
4 (B_1_)	26.89	0.71	35.11	1.63	**2.10**	0.60	0.39	0.21	0.18	-
5 (A_1_)	3.92	0.82	14.72	3.41	0.31	0.25	0.16	0.22	0.33	0.32
6 (A_1_)	12.83	6.49	58.06	17.59	1.00	1.00	1.00	1.00	1.00	1.00
7 (B_2_)	20.08	1.63	72.64	4.16	1.57	1.25	2.07	0.24	0.23	0.19
8 (B_1_)	47.33	3.08	32.67	4.45	**3.69**	0.56	0.53	**0.40**	0.20	0.12
9 (B_2_)	0.00	7.27	303.43	4.86	10.58	5.23	4.85	0.15	0.20	0.20
10 (A_2_)	135.70	1.42	0.00	13.28	-	-	-	**0.83**	**0.54**	
11 (A_1_)	98.17	11.42	218.60	10.75	**7.65**	3.76	1.11	**1.16**	0.41	0.21
12 (B_1_)	133.54	0.24	133.91	0.21	**10.41**	2.31	2.14	0.02	0.01	-
13 (A_1_)	1.82	7.49	6.40	20.09	0.14	0.11	0.10	0.70	0.68	0.54
14 (A_1_)	1.45	48.30	6.71	222.01	0.11	0.12	0.13	2.13	3.50	1.33
15 (B_2_)	2.38	25.19	8.04	85.53	0.19	0.14	0.08	1.09	1.32	1.23
tot	484.39	114.35	900.03	389.47						

**Table 3:** IR intensity (IR, km mol^−1^) and Raman activity (R, Å^4^ mol^−1^) from the computed spectra of the 3D β-crystal of PVDF, while increasing the expansion factor f. (**A**) f describes the isotropic cell expansion in the (**a**, **b**) plane, (**B**) f describes the cell expansion along the **a**-axis. The total intensity (tot) is obtained as the sum of the band intensities over all normal modes; partial contribution due to the sum of intensities of modes belonging to the same symmetry species is also reported.

(**A**) **Isotropic cell expansion in the (a, b) plane → isolated chain = 1D crystal**												
f	1	1.02	1.06	1.1	1.2	1.3	1.4	1.5	1.6	1.8	2	1D
Irrep	IR intensity (km mol^−1^)											
B_2_	404	386	357	331	286	254	235	222	212	199	191	159
B_1_	202	201	200	199	199	200	201	202	203	203	204	207
A_1_	304	292	271	253	219	196	181	170	161	150	143	118
A_2_	0	0	0	0	0	0	0	0	0	0	0	0
Tot	910	879	827	784	704	650	616	593	576	553	539	484
Irrep	Raman activity (Å^4^ amu^−1^)											
B_2_	95	95	92	90	78	65	57	51	47	42	39	28
B_1_	6	6	6	6	6	5	5	5	5	5	4	4
A_1_	274	264	239	222	180	148	130	117	110	100	94	74
A_2_	14	14	13	12	11	11	10	10	9	9	9	8
Tot	389	380	350	330	275	229	202	183	171	156	147	114
(**B**) **Cell expansion along the a-axis → vertical slab**												
f	1	1.02	1.06	1.1	1.2	1.3	1.4	1.5	1.6	1.8	2	1D
Irrep	IR intensity (km mol^−1^)											
B_2_	404	411	357	326	54	45	231	213	200	182	168	159
B_1_	202	175	200	200	199	198	198	198	198	198	198	208
A_1_	304	301	296	294	289	286	285	285	284	284	284	118
A_2_	0	0	0	0	232	0	0	0	0	0	0	0
Tot	910	887	853	820	774	530	714	696	683	664	650	485
Irrep	Raman activity (Å^4^ amu^−1^)											
B_2_	95	94	87	81	76	66	55	50	47	43	40	28
B_1_	6	5	6	7	7	7	7	7	7	7	7	4
A_1_	274	266	246	232	203	182	170	161	156	149	145	75
A_2_	14	13	12	12	5	9	8	7	7	6	6	8
Tot	389	378	352	331	290	264	240	225	216	205	197	114

**Table 4:** Computed values of the dipole derivative associated with the CH stretching modes (Q_14_ and Q_15_) of the PVDF crystal while varying the expansion coefficient f (isotropic expansion in the (**a**, **b**) plane); f ranges from 1 to 2.

Mode	Dipole Derivative (Debye/Å amu^1/2^)	f = 1	1.01	1.06	1.1	1.2	1.3	1.4	1.5	1.6	1.7	1.8	2
14 (A_1_)	$\frac{\partial M^{y}}{\partial Q_{14}}$	−0.40	−0.37	−0.24	−0.17	−0.08	−0.03	0.01	0.04	0.06	0.08	0.09	0.11
15 (B_2_)	$\frac{\partial M^{x}}{\partial Q_{15}}$	−0.44	−0.40	−0.24	−0.16	−0.03	0.03	0.07	0.10	0.12	0.14	0.15	0.17

## Data Availability

Data is contained within the article or supplementary material.

## Supplementary Materials

The following supporting information can be downloaded at https://www.mdpi.com/article/10.3390/ma16176004/s1, Figure S1: (**A**) Infrared spectra of a PVDF film sample and PVDF electrospun fibers; (**B**) Raman spectra of a PVDF film sample and PVDF electrospun fibers. Spectral markers of different PVDF polymorphs are highlighted. Figure S2: Result of curve fitting of the infrared spectrum of PVDF film. Figure S3: Result of curve fitting of the Raman spectrum of PVDF film. Table S1: Wavenumbers, IR intensity, and Raman activities of normal modes of PVDF from the DFT computed spectra of the 3D β-crystal while increasing the expansion factor f.
