# Supplementary material for: Molecular Mechanism of the Piezoelectric Response in the β-Phase PVDF Crystals Interpreted by Periodic Boundary Conditions DFT Calculations
Source: Materials (Basel). 2023 Aug 31;16(17):6004. doi: 10.3390/ma16176004 (PMC10488459; doi:10.3390/ma16176004)
Supplement: Supplementary file 1 [file materials-16-06004-s001.zip › materials-2554129-supplementary.pdf]

Molecular mechanism of the piezoelectric response in the β-phase PVDF crystals interpreted by periodic boundary conditions DFT calculations

Gianluca Serra, Alessia Arrigoni, Mirella Del Zoppo, Chiara Castiglioni*, Matteo Tommasini*

Dipartimento di Chimica, Materiali e Ingegneria Chimica "Giulio Natta", Politecnico di Milano, Piazza Leonardo da Vinci 32, 20133 Milano, Italy

*Corresponding authors: matteo.tommasini@polimi.it; chiara.castiglioni@polimi.it

SUPPLEMENTARY MATERIALS

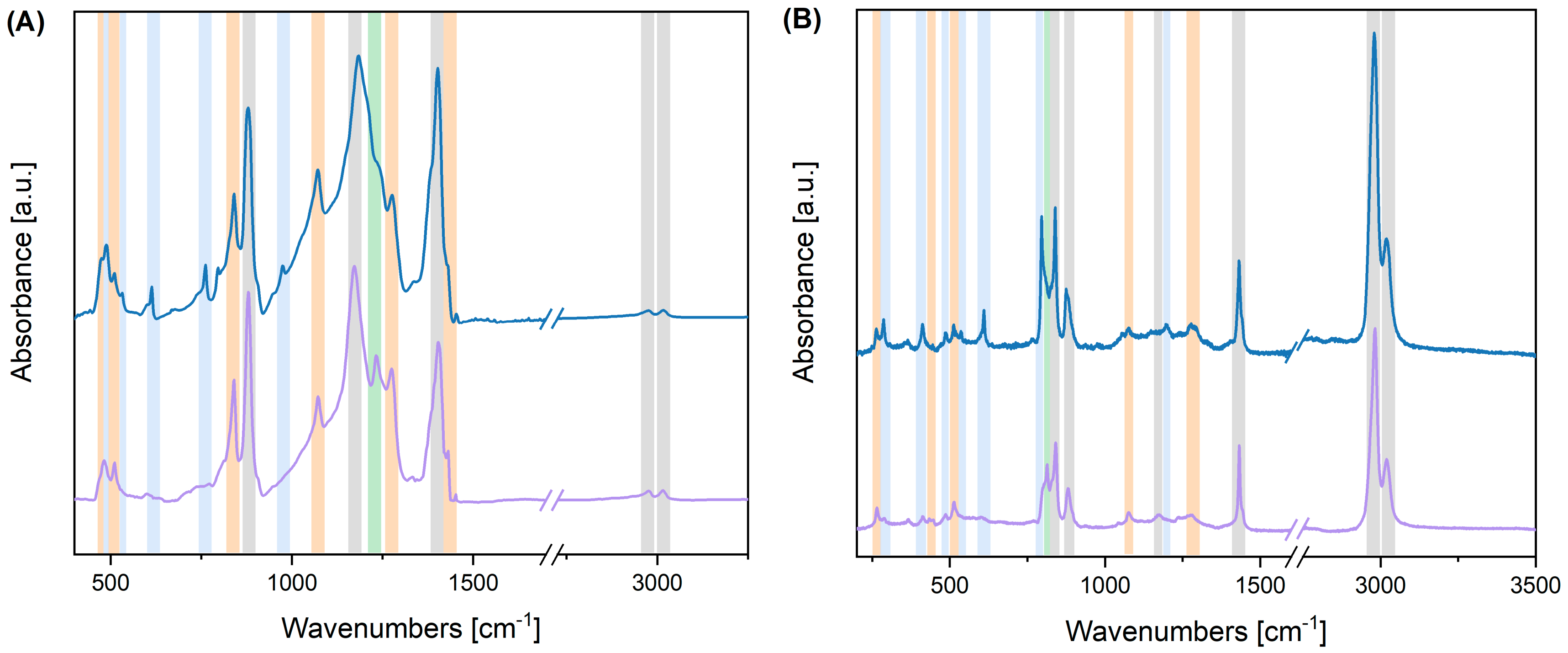

Figure S1: *(A) Infrared spectra of a PVDF film sample (violet line) and PVDF electrospun fibers (blue line). (B) Raman spectra of a PVDF film sample (violet line) and PVDF electrospun fibers (blue line). Spectral markers of different PVDF polymorphs are highlighted: light blue is the α-phase, orange is the β-phase, green is the γ-phase, and grey bands are related to both α- and β-phase. Fibers are reported as a reference sample which shows clear maker bands due to the presence of the α polymorph.*

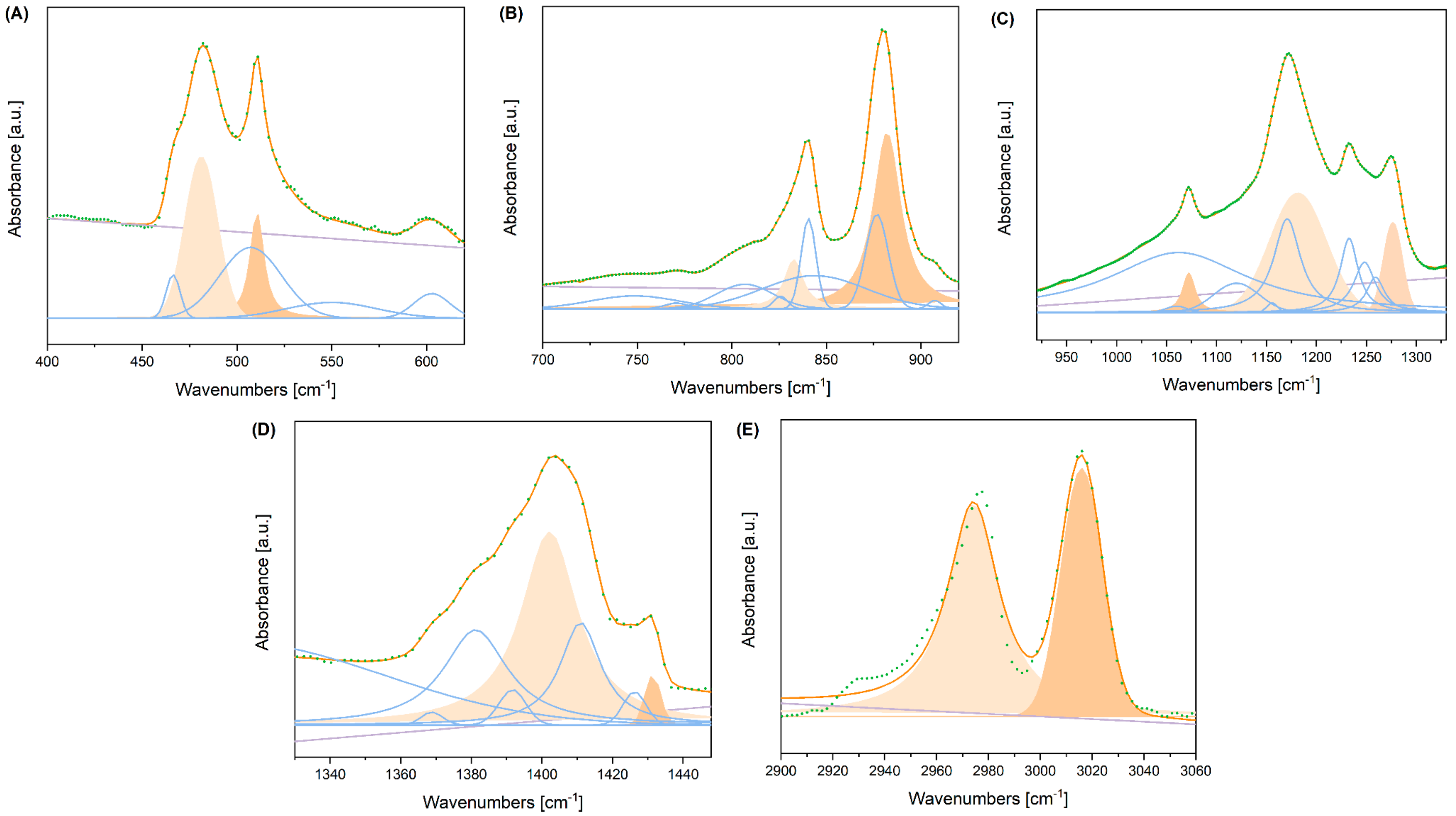

***Figure S2**: Results of the band deconvolution of selected regions of the Infrared spectrum of a PVDF film. The deconvolution was performed by the Fityk software (M. Wojdyr, J. Appl. Cryst. 43, 1126-1128 (2010)). In each panel: green dots are the experimental spectra, the orange line is the reconstructed spectrum, the violet line is the linear baseline, the orange-filled curves are the single components related to the β-phase of PVDF (see main text), and the light blue curves are the other single components.*

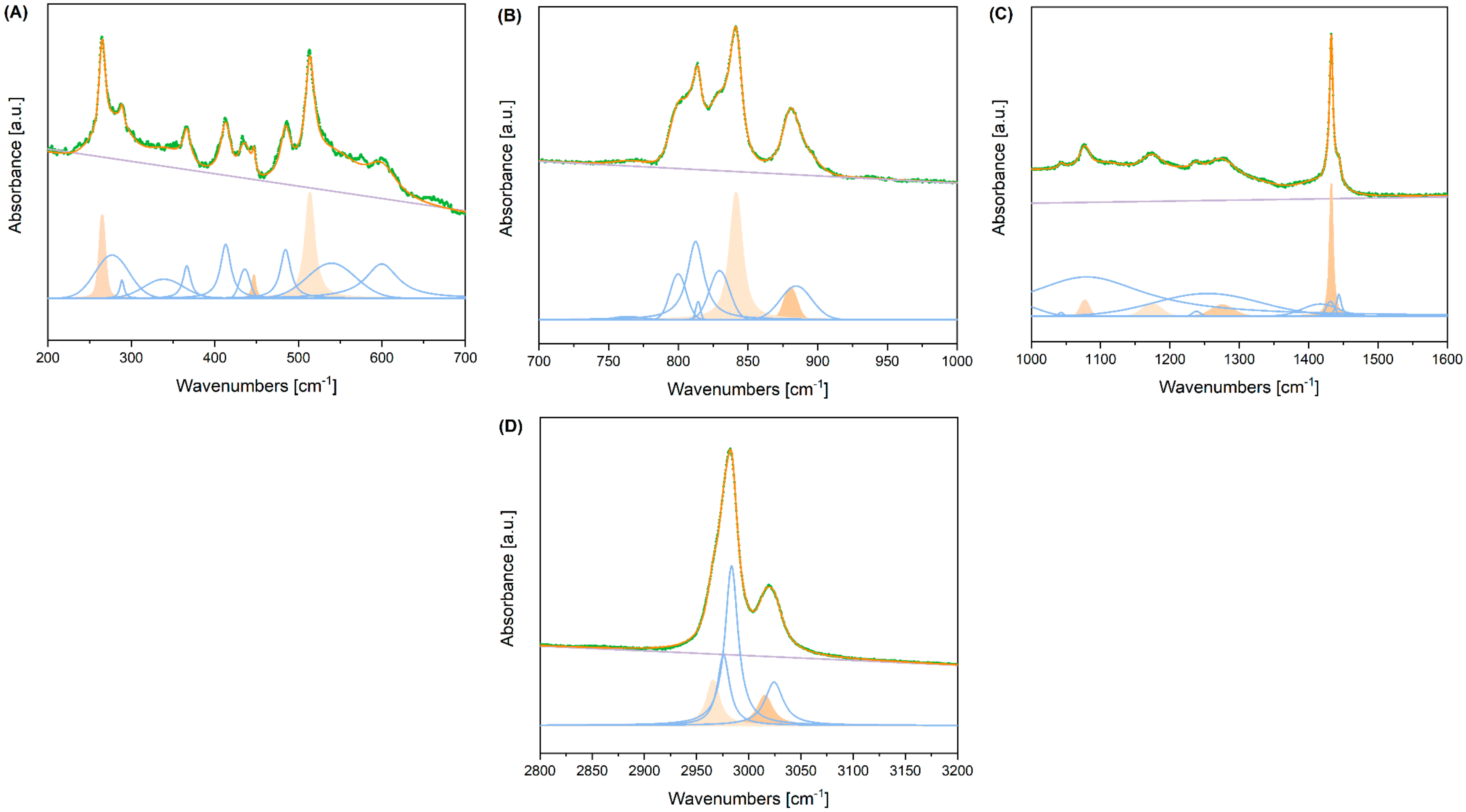

Figure S3*: Results of the band deconvolution of selected regions of the Raman spectrum of a PVDF film. The deconvolution was performed by the Fityk software (M. Wojdyr, J. Appl. Cryst. 43, 1126-1128 (2010)). In each panel: green dots are the experimental spectra, the orange line is the reconstructed spectrum, the violet line is the linear baseline, the orange-filled curves are the single components related to the β-phase of PVDF (see main text), and the light blue curves are the additional components.*

***Table S1**: Wavenumbers, IR intensity (km mol^{-1}), and Raman activities (Å^4 mol^{-1}) of normal modes of PVDF from the DFT computed spectra of the 3D β crystal for increasing values of the expansion factor f. (A) f describes the isotropic cell expansion in the (**a**, **b**) plane, (B) f describes the cell expansion along the **a** axis. The color of the cell background identifies the symmetry species of each mode. A_1 : white, A_2 : yellow, B_1 : blue, B_2 : green. Gray cells correspond to translations.*

(A) ISOTROPIC CELL EXPANSION IN THE (a,b) PLANE → ISOLATED CHAIN = 1D CRYSTAL, f from 1 to 1.3

3D crystal, f=1			1.02			1.06			1.1			1.2			1.3		
Wave number	IR Intensity	Raman Activity	Wave number	IR Intensity	Raman Activity	Wave number	IR Intensity	Raman Activity	Wave number	IR Intensity	Raman Activity	Wave number	IR Intensity	Raman Activity	Wave number	IR Intensity	Raman Activity
-0.06	0.00	0.00	-0.08	0.00	0.00	-0.13	0.00	0.00	-0.32	0.00	0.00	-2.03	0.43	0.00	-13.07	4.19	0.00
-0.05	0.00	0.00	-0.05	0.00	0.00	-0.08	0.00	0.00	-0.09	0.00	0.00	-0.21	0.00	0.00	-0.07	0.00	0.00
-0.04	0.00	0.00	-0.04	0.00	0.00	-0.04	0.00	0.00	-0.03	0.00	0.00	-0.07	0.00	0.00	-0.05	0.00	0.00
74.59	9.79	0.01	53.05	9.17	0.02	29.21	7.84	0.02	19.99	6.65	0.02	6.64	4.58	0.01	0.69	0.01	0.00
260.96	0.00	0.70	260.58	0.00	0.67	259.82	0.00	0.62	259.03	0.00	0.58	258.79	0.00	0.50	258.75	0.00	0.45
444.03	9.73	0.80	432.32	8.21	0.81	426.77	5.91	0.79	428.30	4.26	0.68	429.69	2.32	0.41	429.93	1.54	0.23
452.18	35.11	1.63	451.40	34.26	1.58	450.52	32.95	1.50	450.28	32.03	1.42	450.73	30.76	1.24	451.02	30.12	1.12
513.38	14.72	3.41	512.09	13.92	3.34	511.63	12.54	3.19	511.66	11.42	3.00	512.82	9.50	2.55	512.78	8.26	2.08
861.76	58.06	17.59	862.81	54.72	17.01	864.23	48.63	15.65	866.09	43.64	14.69	870.97	35.44	12.39	873.74	30.02	10.68
903.88	72.64	4.16	904.80	68.50	4.07	904.87	62.62	3.76	905.54	57.63	3.74	909.05	48.41	3.45	912.14	42.26	3.22
1081.11	32.67	4.45	1079.69	33.80	4.44	1077.15	35.41	4.42	1075.24	36.54	4.37	1072.59	38.54	4.18	1070.79	39.82	3.91
1172.95	303.43	4.86	1177.65	295.06	5.01	1187.49	277.84	5.15	1197.16	261.86	4.98	1195.91	0.00	10.97	1193.94	0.00	10.24
1203.67	0.00	13.28	1202.23	0.00	12.89	1200.23	0.00	12.29	1198.41	0.00	11.85	1215.32	230.69	4.07	1227.77	209.78	3.14
1299.09	218.60	10.75	1301.15	212.79	10.83	1305.18	201.59	10.92	1309.24	191.11	10.90	1317.46	169.41	10.85	1323.42	153.73	10.83
1414.84	133.91	0.21	1412.95	132.87	0.25	1409.68	131.58	0.29	1407.51	130.82	0.30	1404.46	129.63	0.24	1402.45	129.81	0.21
1448.25	6.40	20.09	1444.40	5.90	19.24	1444.85	5.62	17.95	1447.27	5.36	17.05	1451.92	4.84	15.57	1454.69	4.21	13.16
3108.41	6.71	222.01	3104.58	4.64	213.90	3095.20	2.43	191.76	3091.00	1.28	176.34	3090.51	0.25	138.27	3092.39	0.03	111.36
3166.68	8.04	85.53	3162.51	5.28	85.59	3151.99	2.49	82.08	3147.65	1.05	80.53	3146.43	0.03	69.89	3148.02	0.05	58.80

(A) ISOTROPIC CELL EXPANSION IN THE (**a**,**b**) PLANE → ISOLATED CHAIN = 1D CRYSTAL, f from 1.4 to 2

1.4			1.5			1.6			1.8			2			single chain (1D crystal)		
Wave number	IR intensity	Raman Activity	Wave number	IR intensity	Raman Activity	Wave number	IR intensity	Raman Activity	Wave number	IR intensity	Raman Activity	Wave number	IR intensity	Raman Activity	Wave number	IR intensity	Raman Activity
-20.92	3.69	0.00	-21.28	3.34	0.00	-49.22	3.04	0.00	-10.08	2.65	0.00	-2.05	0.80	0.00	-8.53	2.02	0.00
-0.11	0.00	0.00	-0.23	0.00	0.00	-5.82	0.00	0.00	-0.29	0.00	0.00	-0.06	0.00	0.00	-0.06	0.00	0.00
-0.03	0.00	0.00	-0.03	0.00	0.00	-0.04	0.00	0.00	-0.05	0.00	0.00	-0.05	0.00	0.00	-0.05	0.00	0.00
0.80	0.01	0.00	0.89	0.01	0.00	8.16	0.10	0.00	2.39	0.15	0.00	3.09	1.83	0.00	1.99	0.11	0.00
258.84	0.00	0.42	259.22	0.00	0.40	259.64	0.00	0.38	259.76	0.00	0.35	259.89	0.00	0.34	260.33	0.00	0.28
430.25	1.08	0.15	430.93	0.81	0.10	424.25	0.56	0.07	430.37	0.51	0.05	430.96	0.44	0.04	428.68	0.29	0.02
451.22	29.71	1.04	451.79	29.32	0.98	452.29	28.99	0.94	452.37	28.49	0.88	452.77	28.20	0.84	453.45	26.89	0.71
512.73	7.40	1.76	513.34	6.77	1.55	513.81	6.27	1.40	513.57	5.66	1.25	513.59	5.25	1.14	513.52	3.92	0.82
876.09	26.21	9.65	878.30	23.72	8.91	883.19	22.07	8.31	882.12	19.93	7.92	881.81	18.03	7.61	884.09	12.83	6.49
914.68	37.77	3.03	916.93	34.63	2.80	924.03	34.40	2.69	920.34	29.27	2.38	921.86	27.29	2.22	926.83	20.08	1.63
1069.50	40.74	3.72	1068.53	41.30	3.61	1067.62	41.63	3.52	1066.18	42.41	3.41	1065.20	42.84	3.33	1058.09	47.33	3.08
1192.93	0.00	9.69	1191.87	0.00	9.29	1191.21	0.00	9.00	1190.00	0.00	8.60	1189.09	0.00	8.32	1185.42	0.00	7.27
1237.01	195.74	2.58	1243.94	185.81	2.30	1250.77	176.32	2.09	1254.76	167.73	1.95	1258.56	160.62	1.83	1273.40	135.70	1.42
1327.53	143.24	10.89	1331.37	135.83	10.97	1327.76	132.88	12.47	1339.17	121.52	11.04	1337.42	116.76	11.08	1341.83	98.17	11.42
1400.83	130.45	0.21	1399.46	131.18	0.22	1397.87	131.94	0.23	1395.94	132.56	0.23	1394.58	133.08	0.24	1384.57	133.54	0.24
1456.17	3.66	11.38	1457.16	3.23	10.33	1445.62	0.12	8.39	1456.71	2.98	9.19	1456.89	2.63	8.73	1456.68	1.82	7.49
3093.76	0.00	95.84	3094.77	0.06	85.60	3119.12	0.15	79.31	3094.08	0.37	71.08	3093.23	0.55	65.93	3090.81	1.45	48.30
3149.51	0.20	51.45	3150.70	0.41	46.14	3149.22	0.60	42.56	3150.90	0.97	38.04	3150.53	1.23	35.13	3148.57	2.38	25.19

(B) CELL EXPANSION ALONG THE **a** AXIS → VERTICAL SLAB, f from 1 to 1.3

3D crystal, f=1			1.02			1.06			1.1			1.2			1.3		
Wave number	IR intensity	Raman Activity	Wave number	IR intensity	Raman Activity	Wave number	IR intensity	Raman Activity	Wave number	IR intensity	Raman Activity	Wave number	IR intensity	Raman Activity	Wave number	IR intensity	Raman Activity
-0.06	0.00	0.00	-0.06	0.00	0.00	-0.07	0.00	0.00	-10.35	7.52	0.01	-0.45	0.00	0.00	-0.51	0.00	0.00
-0.05	0.00	0.00	-0.06	0.00	0.00	-0.04	0.00	0.00	-0.10	0.00	0.00	-0.09	0.00	0.00	-0.11	0.00	0.00
-0.04	0.00	0.00	-0.04	0.00	0.00	-0.04	0.00	0.00	-0.03	0.00	0.00	-0.03	0.00	0.00	-0.03	0.00	0.00
74.59	9.79	0.01	78.78	8.46	0.01	52.64	7.93	0.02	0.91	0.06	0.00	27.44	5.76	0.01	32.09	4.88	0.00
260.96	0.00	0.70	261.03	0.00	0.67	260.90	0.00	0.63	260.13	0.00	0.58	259.83	0.00	0.50	259.70	0.00	0.44
444.03	9.73	0.80	452.11	34.72	1.62	442.58	6.78	0.88	430.83	5.60	0.65	436.59	3.80	0.54	436.18	2.93	0.44
452.18	35.11	1.63	455.84	8.44	0.78	451.69	33.99	1.60	451.31	33.51	1.59	451.62	32.80	1.57	451.85	32.54	1.55
513.38	14.72	3.41	514.02	14.47	3.34	512.82	14.47	3.29	512.16	14.85	3.34	513.32	14.89	3.17	513.71	14.93	3.03
861.76	58.06	17.59	863.01	57.03	17.19	864.27	55.37	16.47	863.49	54.38	15.67	866.03	51.80	14.62	866.99	50.63	13.97
903.88	72.64	4.16	905.76	70.60	4.06	908.23	61.92	3.50	906.50	55.21	3.39	908.88	44.14	2.88	910.04	37.25	2.57
1081.11	32.67	4.45	1080.87	33.10	4.45	1080.04	34.35	4.58	1078.82	34.99	4.66	1077.74	35.81	4.80	1077.10	36.28	4.86
1172.95	303.43	4.86	1175.53	291.88	4.79	1178.56	277.33	4.99	1180.81	263.63	4.72	1189.94	232.21	4.41	1196.02	208.26	3.95
1203.67	0.00	13.28	1202.95	0.00	12.67	1202.97	0.00	11.74	1201.92	0.00	10.97	1200.74	0.00	9.51	1199.99	0.00	8.46
1299.09	218.60	10.75	1299.74	216.78	10.67	1301.49	214.88	10.72	1302.59	213.80	10.70	1305.61	211.36	10.69	1307.35	210.20	10.73
1414.84	133.91	0.21	1414.16	133.51	0.22	1413.11	131.97	0.24	1412.02	131.19	0.25	1410.90	130.04	0.26	1410.33	129.47	0.26
1448.25	6.40	20.09	1455.09	7.04	19.71	1452.50	7.36	18.87	1448.19	7.41	18.85	1454.55	8.31	17.58	1456.99	8.78	16.61
3108.41	6.71	222.01	3109.10	5.65	214.64	3103.39	4.09	196.64	3097.80	3.38	183.69	3099.28	2.24	156.66	3100.87	1.81	137.93
3166.68	8.04	85.53	3167.45	5.73	83.19	3160.79	2.95	77.52	3154.78	1.84	72.40	3155.36	0.56	62.71	3156.07	0.32	54.52

(B) CELL EXPANSION ALONG THE **a** AXIS → VERTICAL SLAB, f from 1.4 to 2

1.4			1.5			1.6			1.8			2			single chain (1D crystal)		
Wave number	IR intensity	Raman Activity	Wave number	IR intensity	Raman Activity	Wave number	IR intensity	Raman Activity	Wave number	IR intensity	Raman Activity	Wave number	IR intensity	Raman Activity	Wave number	IR intensity	Raman Activity
-0.85	0.00	0.00	-7.41	0.07	0.00	-2.69	0.00	0.00	-0.63	0.00	0.00	-0.61	0.00	0.00	-8.53	2.02	0.00
-0.08	0.00	0.00	-1.51	0.00	0.00	-0.78	0.00	0.00	-0.12	0.00	0.00	-0.10	0.00	0.00	-0.06	0.00	0.00
-0.04	0.00	0.00	-0.09	0.00	0.00	-0.04	0.00	0.00	-0.12	0.00	0.00	-0.03	0.00	0.00	-0.05	0.00	0.00
27.58	4.36	0.00	53.91	3.86	0.00	34.26	3.56	0.00	36.86	3.12	0.00	39.06	2.84	0.00	1.99	0.11	0.00
259.56	0.00	0.41	259.69	0.00	0.38	259.84	0.00	0.35	259.87	0.00	0.32	259.92	0.00	0.30	260.33	0.00	0.28
435.02	2.47	0.39	436.11	2.19	0.29	436.61	1.93	0.28	437.25	1.67	0.24	437.36	1.51	0.22	428.68	0.29	0.02
451.87	32.44	1.55	452.07	32.38	1.54	446.51	31.39	1.57	452.16	32.25	1.53	452.27	32.25	1.53	453.45	26.89	0.71
513.44	14.88	2.87	512.94	14.88	2.74	513.83	14.88	2.63	513.94	14.87	2.51	514.07	14.86	2.43	513.52	3.92	0.82
867.25	50.23	13.52	866.24	49.30	13.19	867.53	49.77	12.96	867.92	49.84	12.69	867.71	49.53	12.53	884.09	12.83	6.49
910.52	32.54	2.34	908.78	28.29	2.15	911.57	26.88	2.04	912.62	23.59	1.84	913.19	21.30	1.70	926.83	20.08	1.63
1076.78	36.44	4.88	1076.67	36.60	4.89	1076.18	37.46	4.87	1076.56	36.80	4.90	1076.49	36.80	4.90	1058.09	47.33	3.08
1198.84	0.00	7.68	1198.73	0.00	7.09	1198.57	0.00	6.66	1198.35	0.00	6.03	1198.38	0.00	5.59	1185.42	0.00	7.27
513.44	14.88	2.87	512.94	14.88	2.74	513.83	14.88	2.63	513.94	14.87	2.51	514.07	14.86	2.43	513.52	3.92	0.82
1200.25	190.92	3.61	1203.48	178.78	3.37	1206.56	167.83	3.21	1211.05	152.99	3.02	1214.35	142.54	2.89	1273.40	135.70	1.42
1307.99	209.38	10.74	1309.09	207.79	10.53	1308.38	208.88	10.76	1309.75	208.50	10.78	1308.56	208.64	10.78	1341.83	98.17	11.42
1410.34	129.40	0.26	1410.20	129.35	0.26	1408.10	129.44	0.25	1410.00	129.20	0.26	1410.28	129.20	0.26	1384.57	133.54	0.24
1458.33	8.98	15.86	1462.64	10.98	15.36	1460.03	9.28	14.89	1460.68	9.32	14.51	1460.51	9.38	14.27	1456.68	1.82	7.49
3103.18	1.67	126.67	3104.85	1.59	118.72	3105.44	1.52	114.27	3105.54	1.46	108.53	3105.32	1.45	104.92	3090.81	1.45	48.30
